# Phylogenetic Responses of Forest Trees to Global Change

**DOI:** 10.1371/journal.pone.0060088

**Published:** 2013-04-04

**Authors:** John K. Senior, Jennifer A. Schweitzer, Julianne O’Reilly-Wapstra, Samantha K. Chapman, Dorothy Steane, Adam Langley, Joseph K. Bailey

**Affiliations:** 1 School of Plant Science, University of Tasmania, Hobart, TAS, Australia; 2 Department of Ecology and Evolutionary Biology, University of Tennessee, Knoxville, Tennessee, United States of America; 3 School of Plant Science and National Centre for Future Forest Industries, University of Tasmania, Hobart, TAS, Australia; 4 Department of Biology, Villanova University, Villanova, Pennsylvania, United States of America; 5 University of the Sunshine Coast, Sippy Downs, Queensland, Australia; Lakehead University, Canada

## Abstract

In a rapidly changing biosphere, approaches to understanding the ecology and evolution of forest species will be critical to predict and mitigate the effects of anthropogenic global change on forest ecosystems. Utilizing 26 forest species in a factorial experiment with two levels each of atmospheric CO_2_ and soil nitrogen, we examined the hypothesis that phylogeny would influence plant performance in response to elevated CO_2_ and nitrogen fertilization. We found highly idiosyncratic responses at the species level. However, significant, among-genetic lineage responses were present across a molecularly determined phylogeny, indicating that past evolutionary history may have an important role in the response of whole genetic lineages to future global change. These data imply that some genetic lineages will perform well and that others will not, depending upon the environmental context.

## Introduction

Elevated carbon dioxide (CO_2_) and nitrogen (N) availability are expected to have important consequences for forest ecosystem dynamics [1]–[4], where soil N scarcity may limit the CO_2_ fertilisation effect [5], [6]. Atmospheric CO_2_ concentrations are expected to double by the turn of the century [3], while anthropogenic N fixation rates have already doubled preindustrial rates [7], [8]. Tree species have vital roles in carbon (C) and N cycling and are expected to act as important sinks for anthropogenic CO_2_ emissions [2]. As the dominant constituents of forest ecosystems, tree species also have important extended community effects such as plant-plant and plant-herbivore interactions [9], [10]. It is, therefore, vital that we understand the consequences of anthropogenic CO_2_ and N fertilisation on tree species to predict future impacts of these important global change factors on forest ecosystems.

A recent direction in global climate change research is the utilization of phylogenetics to better understand and predict the impacts of global change [11]–[15]. Closely related taxa have the potential to respond in a similar manner to global environmental changes, due to shared evolutionary histories, genetic background, and phenotypic traits. Thus, taking phylogeny into account may provide generality that is more appropriate for modelling the impacts of large-scale global climate change than generalising across species that share fundamental niches. For example, Davis *et al.* [14] assessed the flowering time of plant clades occurring in both the United States and the United Kingdom and found that phenological responses to global climate change were shared within clades. Similar trends are likely to occur in the responses of other plant traits to other large-scale perturbation. For example, the magnitude of CO_2_-induced increases in biomass may vary much more within functional types (e.g. herbaceous vs woody species) than among them [16] though consistent differences in response may arise when functional groups align with major phylogenetic differences such as gymnosperms vs. angiosperms [17]. However, few studies utilise an explicit phylogenetic framework (see [11], [13]–[15]) to assess the importance of phylogeny. Such an approach is important as the differences amongst plant functional groups that are currently being explored in global change studies, likely represent the evolutionary consequences of phylogenetic divergence [18]–[20]. Examining phylogenetic responses to climate change may capture a broader range of variation [21] among taxa for better understanding how plants can respond to increasing CO_2_, N or other environmental factors.

If a phylogenetic approach is useful to understanding the consequences of global change, *Eucalyptus* represents a model genus in which to test it. *Eucalyptus* is a globally important forest plantation species that is planted worldwide and is the dominant genus in many Australian ecosystems, occurring in subalpine woodlands, cool and warm temperate forests, rainforests and tropical savannahs [22]. Having evolved under a large range of climatic and edaphic conditions the genus is also highly diverse with over 700 species displaying a wide range of growth forms, from giant forest to dwarf coastal trees and stunted, multi-stemmed, “mallee” forms in semi-arid areas [23]. This genus is of great ecological and economic importance, yet relatively few studies have been concerned with the effects of climate change on eucalypt forests in native or non-native habitats around the world. Studies to date have found that eucalypt species are responsive to elevated CO_2_ and N fertilization but responses differ in direction and magnitude, with negative, neutral and positive growth responses documented [24]–[30]. Consequently, the response of eucalypt species to elevated CO_2_ and N fertilization may not be as general as expected and a phylogenetic approach may be useful to better inform responses to global change.

On the island of Tasmania (Australia), there are 29 species of *Eucalyptus* that occur in a range of habitats from coastal wet and dry forests to alpine environments. The species belong to the two main subgenera of *Eucalyptus* (i.e., subgenus *Symphyomyrtus* and subgenus *Eucalyptus*; [23]), but lower taxonomic classifications are unresolved, with a number of authors grouping species in different ways [23], [31], [32]. In this paper we follow the classification of Brooker [23] in which the classification of eucalypt species is based predominantly on morphological traits, including bark, leaf, floral and fruit morphologies, and is largely supported by the available molecular data [33], [34].

Using 26 of the 29 species of *Eucalyptus* found in Tasmania, we used a phylogenetic approach to better understand plant performance in response to the global change factors of elevated CO_2_ and soil N fertilization. All species were exposed to factorial treatments of ambient and elevated CO_2_ and low and high soil N concentrations, where it was hypothesised that plant performance of closely related species to these two global change factors would be similar. Specifically, we hypothesized that eucalypt species would respond differentially to elevated CO_2_ and N fertilization based on past, shared evolutionary history. We found that; 1) individual species’ growth responses are largely idiosyncratic, however, 2) when species were nested within genetic lineages, species within a particular genetic lineage shared similar responses to elevated CO_2_ and high soil N concentrations, significantly differing from other genetic lineages. Utilising a phylogenetic approach may, therefore, provide a potential framework by which the responses of individual forest species to global climate change may be generalized across groups of closely related species. These results overall suggest that there may be phylogenetic “winners” and “losers” in response to global change factors based on past evolutionary dynamics and/or history.

## Methods

### Plant Material

Seed of 26 native Tasmanian eucalypt species was purchased from Forestry Tasmania (http://www.forestrytas.com.au/); *E. archeri*, *E. morrisbyi* and *E. coccifera,* were not included in the study because seed was not available. Seed of each species was obtained from one to six individual maternal trees from a single population. To enhance germination, the seed of each species was vernalised before sowing. Seed of each species was folded in paper towel, wrapped in cheese cloth and soaked in a solution of water containing a drop of dishwashing detergent overnight. Each seed bundle was then squeezed the following morning to remove excess water and refrigerated for 30 days at 4°C. After this period, the seed of each species was dispersed on the surface of 26 separate trays filled with commercial potting mix, which consisted of eight parts composted fine pine bark and three parts coarse river sand with added macro- and micro-nutrients from Nutricote Grey (Langley Australia Pty Ltd., Welshpool WA), which included N, phosphorus (P) and potassium (K) in the weight ratio of 19∶2.6∶10, at a rate of 3 kg/m^−3^. The surface of the potting mix was then disturbed gently and covered with vermiculite for water retention. The trays received a daily soaking of water, in equal volumes, in a greenhouse while seeds germinated. Germinants were grown for three weeks until the majority of seedlings of each species had developed the first pair of true leaves and were uniform in size.

### Phylogenetic Framework for Tasmanian Eucalypts

A phylogenetic framework for this study was devised using the classification of Brooker [23] with adjustments using recent molecular genetic information on the subgenera *Symphyomyrtus* [33] and *Eucalyptus* [34], [35]. Hence, we have devised a recent, genetically informed taxonomical classification. The Tasmanian species belonging to subgenus *Eucalyptus* were placed in Genetic Lineage 1 (GL1). The four Tasmanian species of series *Foveolatae* (*E. barberi*, *E. brookeriana, E. ovata,* and *E. rodwayi*) formed Genetic Lineage 2 (GL2); Tasmanian endemics belonging to series *Orbiculares* and *Semiunicolores* formed GL3. The remaining (non-endemic) species of subgenus *Symphyomyrtus* (*E. globulus*, series *Globulares*; *E. perriniana*, series *Orbiculares*; *E. viminalis*, *E. rubida* and *E. dalrympleana*, series *Viminales*) were place in GL4. These genetic lineages were used to conduct nested analyses to test for the effects of phylogenetic group and subgenus (Table 1).

**Table 1:** Phylogenetic classification of all Tasmanian eucalypt species based on Brooker [23] and DArT data from this study, informed with recent genetic data from McKinnon *et al.* [33] and Steane *et al.* [34].

Subgenus	Genetic lineage^1^	Species	Section/Subsection^2^, Series Brooker [21]	Code^3^
		*E. obliqua*	*Eucalyptus, Eucalyptus*	*EEE*
		*E. regnans*	*Eucalyptus, Regnantes*	*EER*
		*E. sieberi*	*Cineracea, Psathyroxyla*	*ECPS*
		*E. delegatensis*	*Cineracea, Fraxinales*	*ECF*
		*E. pauciflora*	*Cineracea, Pauciflorae*	*ECP*
*Eucalyptus*	Genetic lineage 1	*E. radiata*	*Aromatica, Radiatae*	*EAR*
		*E. amygdalina*	*Aromatica, Insulanae*	*EAI*
		*E. nitida*	*Aromatica, Insulanae*	*EAI*
		*E. pulchella*	*Aromatica, Insulanae*	*EAI*
		*E. risdonii*	*Aromatica, Insulanae*	*EAI*
		*E. tenuiramis*	*Aromatica, Insulanae*	*EAI*
		*E. brookeriana*	*Triangulares, Foveolatae*	*SMTF*
	Genetic lineage 2	*E. ovata*	*Triangulares, Foveolatae*	*SMTF*
		*E. rodwayi*	*Triangulares, Foveolatae*	*SMTF*
		*E. barberi*	*Triangulresa, Foveolatae*	*SMTF*
		*E. archeri* 4	*Euryota, Orbiculares*	*SMEO*
		*E. cordata*	*Euryota, Orbiculares*	*SMEO*
		*E. gunnii*	*Euryota, Orbiculares*	*SMEO*
	Genetic lineage 3	*E. morrisbyi* 4	*Euryota, Orbiculares*	*SMEO*
*Symphyomyrtus*		*E. urnigera*	*Euryota, Orbiculares*	*SMEO*
		*E. johnstonii*	*Euryota, Semiunicolores*	*SMES*
		*E. subcrenulata*	*Euryota, Semiunicolores*	*SMES*
		*E. vernicosa*	*Euryota, Semiunicolores*	*SMES*
		*E. globulus*	*Euryota, Globulares*	*SMEG*
		*E. perriniana*	*Euryota, Orbiculares*	*SMEO*
	Genetic lineage 4	*E. dalrympleana*	*Euryota, Viminales*	*SMEV*
		*E. rubida*	*Euryota, Viminales*	*SMEV*
		*E. viminalis*	*Euryota, Viminales*	*SMEV*

1 “Genetic lineage” designation is based on AFLP [33] and/or DArT analyses [34], [35].

2 Sectional classification is given for species belonging to subgenus *Eucalyptus* (no sub-sectional classification available); all Tasmanian species from subgenus *Symphyomyrtus* belong to section *Maidenaria*, so only subsections are shown here.

3 Taxonomic code representing subgenus, section, subsection (for subgenus *Symphyomyrtus* only) and series.

4 No DArT data available for these species.

Further support for these groupings was found in broad analyses of all eucalypt subgenera (that did not include all the Tasmanian species that were included in this study; [34], [35]) that used a relatively new molecular marker called Diversity Arrays Technology (DArT; [36]). In this study, we used DArT to check the genetic integrity of the groupings that we defined and found that genotyping supported the genetic lineages (see [34]).

### Elevated CO_2_ and Soil N Fertilization Study

To determine responses to the global change factors of elevated CO_2_ and soil N fertilization, eucalypt seedlings were grown under all factorial combinations of ambient and elevated CO_2_ and high and low soil N fertilization. Twelve seedlings of each species were transplanted into forestry tubes filled with the same commercial potting mix (described above). These twelve seedlings of each species were divided randomly into three replicates for each of the four factorial treatments of CO_2_ (ambient or elevated) and soil N (low and high). The seedlings for each level of CO_2_ were then placed randomly, via random number generation, into forestry tube racks.

Seedling racks for each CO_2_ treatment were placed randomly into separate, air-tight, controlled greenhouse chambers maintained at 23°C with a natural photoperiod; half of which were then fertilized with N at the soil surface (details below). The CO_2_ treatments and their respective seedlings were exchanged between two greenhouse chambers each week; greenhouse chamber effects were avoided further by moving the CO_2_ tanks and regulator as well as monitoring CO_2_ concentrations with an infra-red gas analyser (IRGA) device (LiCor 6200, LiCor Inc., Lincoln, NE, USA). The forestry tube racks were also repositioned randomly during these periods to avoid seedling positional effects in the greenhouse. In the elevated CO_2_ treatment carbon dioxide was elevated to 720 ppm using a CO_2_ control unit (Thermoline Scientific Equipment, Smithfield, Australia) and compressed CO_2_. The low CO_2_ treatment was maintained at ambient CO_2_ (∼400 ppm) and monitored frequently for leakage of CO_2_ from the neighbouring high CO_2_ chamber with the LiCor (none occurred). Seedlings in each treatment were watered on a daily basis until the eighth week of the study, at which time water was applied as needed. At the same time that the CO_2_ treatments were initiated, pellets of urea at an approximate concentration of 30 kg N ha^−1^, were applied each month to the high N treatment seedlings to replicate the approximate N addition of forestry practices that alleviates N limitation [37].

At the end of the experiment (approximately 5 months later; before seedlings became root bound) each seedling was harvested destructively by carefully removing each seedling, gently shaking off as much soil as possible, and severing the aboveground from the belowground biomass at the root collar. The belowground biomass of each individual of each species was sealed in separate plastic bags after collection and the aboveground biomass was placed in separate brown paper bags. The belowground biomass was refrigerated (6°C) and the aboveground biomass was stored at ambient conditions until oven-drying (at the end of the harvest). The aboveground biomass samples were oven-dried for 48 hours at 60°C and then weighed (g). The belowground biomass of each seedling was carefully rinsed, separately, over 2 and 0.5 mm sieves to remove as much soil as possible whilst retaining as much of the fine root biomass as possible. The washed belowground biomass samples were then oven-dried for 48 hours at 60°C and weighed (g). Oven-dried belowground biomass was divided by aboveground biomass to yield root:shoot and above- and belowground biomass was summed to determine total biomass.

### Statistical Analysis

All statistical analyses were conducted using the statistical package SAS (version 9.2, SAS Institute Inc., Cary USA). Other traits were measured (height, leaf biomass, leaf area and relative growth rate) but were not included in the analysis due to strong inter-correlations with all traits (R^2^>0.7; data not presented). Separate analyses were run for each phylogenetic level to maintain statistical power. The responses of seedling aboveground biomass, belowground biomass, total biomass and root:shoot to elevated CO_2_ and soil N fertilization were analysed to determine how each eucalypt species responded to these environmental variables. Due to mortalities in the greenhouse, whole treatment groups within particular species were absent, making analysis of these species impossible. Consequently, these species were removed from the analysis. There was a highly significant effect of subgenus on species mortality (P<0.001), where 54 seedlings were lost from subgenus *Eucalyptus* and 31 from subgenus *Symphyomyrtus*. Five species were removed from subgenus *Eucalyptus* (GL1) and one species was removed from subgenus *Symphyomyrtus* (GL4) due to the death of whole treatments (n = 3). However, no significant interactions between mortality and environmental treatments were present. General linear models were used to analyse species, CO_2_ (2 levels of treatment, ambient and elevated) and N (2 levels of treatment, low and high) effects for each morphological variable (total biomass, aboveground biomass, belowground biomass, and root:shoot; PROC GLM). All interaction terms were included; species × CO_2_, species × N, CO_2_ × N and species × CO_2_ × N, where all main effects and interaction terms were treated as fixed effects. Data were tested for the assumptions of normality and homoscedasticity and appropriate transformations were applied to meet the Shapiro-Wilk test when required. Diagnostic graphical representations were also checked for normality and homoscedasticity. Aboveground, belowground and total biomass data were square root transformed while root:shoot data were power transformed (0.3). A power transformation was applied when log or square-root transformed data did not satisfy the assumptions of normality and homoscedasticity.

To test for phylogenetic patterns in the responses of the eucalypt species, mixed models were conducted in SAS (PROC MIXED). Models tested genetic lineage (four genetic lineages), CO_2_ (two levels of treatment, ambient and elevated) and N fertilization (two levels of treatment, low and high) effects for each variable (total biomass, aboveground biomass, belowground biomass and root:shoot). These main effects were fixed effects while species(genetic lineage) was used as a random term to test the genetic lineage effect. All interaction terms were also included as fixed effects; genetic lineage × CO_2_, genetic lineage × N, CO_2_ × N and genetic lineage × CO_2_ × N.

To test for the effects of subgenus, CO_2_ and N fertilization, mixed models were used to test for subgenus (two subgenera; *Symphyomyrtus* and *Eucalyptus*), CO_2_ (two levels of treatment, ambient and elevated) and soil N fertilization (two levels of treatment, low and high) effects for each variable (aboveground biomass, belowground biomass, total biomass and root:shoot). These main effects were treated as fixed effects while species(subgenus) was used as a random term to test the subgenus effect. Species(subgenus) was chosen over genetic lineage(subgenus) as this random term conserves a larger proportion of variation. All interaction terms were included; subgenus×CO_2_, subgenus×N, CO_2_×N and subgenus×CO_2_×N as fixed effects.

## Results

### Species Analyses

**Figure 1:**
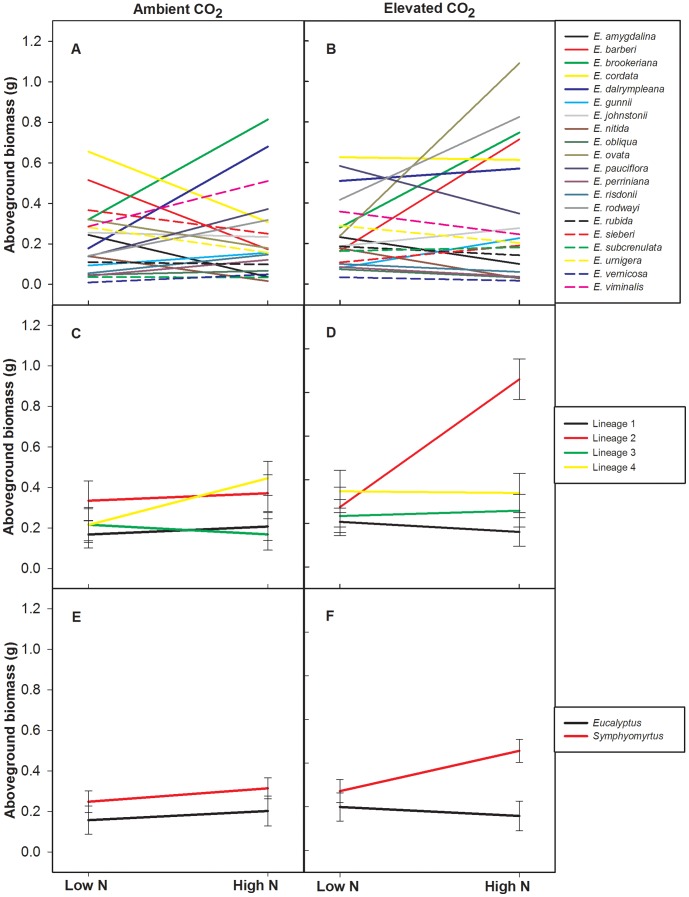
Aboveground biomass responses to elevated CO_2_ and N. Interaction plots of least squares means (± standard error for C–F) of the aboveground biomass of species (A and B), genetic lineages (C and D) and subgenera (E and F) with control soil N and high soil N (30 kg ha^−1^ of N added) under both ambient (left panels) and elevated CO_2_ (right panels). Standard errors are not presented on the species panels (A–B) due to space constraints. Each colour and line (solid/dashed) combination represents each species, genetic lineage or subgenus analysed, which are represented in the respective legends.

There was a three-way interaction between CO_2_, N fertilization and species for aboveground biomass, indicating that the response of plant species to the combination of CO_2_ and soil N fertilization is variable (**Table 2** **;** **Figure 1A, B**). For example, N addition resulted in a nearly three-fold increase in the aboveground biomass of *E. dalrympleana* under ambient CO_2_, whereas under elevated CO_2_, N addition had less impact, resulting only in a 12% increase in aboveground biomass. In contrast, N addition under ambient CO_2_ resulted in a 78% decrease in the aboveground biomass of *E. ovata*, but a large, 3.5-fold, increase under elevated CO_2_.

**Table 2:** General linear model results of variation in aboveground biomass (AGB), belowground biomass (BGB), total biomass (TB) and root:shoot (R:S) between the factors of species, carbon dioxide (CO_2_), nitrogen (N) and all interactions.

	*Species*		*CO_2_*		*N*		Species*CO_2_		Species*N		CO_2_*N		Species*CO_2_*N	
Variable														
	*F*	*P*	*F*	*P*	*F*	*P*	*F*	*P*	*F*	*P*	*F*	*P*	*F*	*P*
**AGB**	7.83_(19,193)_	**<0.001**	2.87_(1,193)_	0.093	1.31_(1,193)_	0.254	0.97_(19,193)_	0.502	1.35_(19,193)_	0.167	0.34_(1,193)_	0.560	1.96_(19,193)_	**0.016**
**BGB**	7.14_(20,198)_	**<0.001**	6.25_(1,198)_	**0.014**	0.33_(1,198)_	0.565	1.01_(20,198)_	0.458	1.12_(20,198)_	0.336	0.11_(1,198)_	0.740	1.85_(20,198)_	**0.023**
**TB**	7.28_(19,190)_	**<0.001**	4.34_(1,190)_	**0.040**	0.57_(1,190)_	0.452	0.89_(19,190)_	0.593	1.18_(19,190)_	0.288	0.34_(1,190)_	0.563	1.92_(19,190)_	**0.019**
**R:S**	3.33_(19,188)_	**<0.001**	8.87_(1,188)_	**0.004**	3.27_(1,188)_	0.073	1.73_(19,188)_	**0.042**	0.46_(19,188)_	0.973	0.12_(1,188)_	0.732	1.91_(19,188)_	0.570

Bold, underlined values indicate statistical significance (α = 0.05); degrees of freedom are denoted as subscript of each F value.

**Figure 2:**
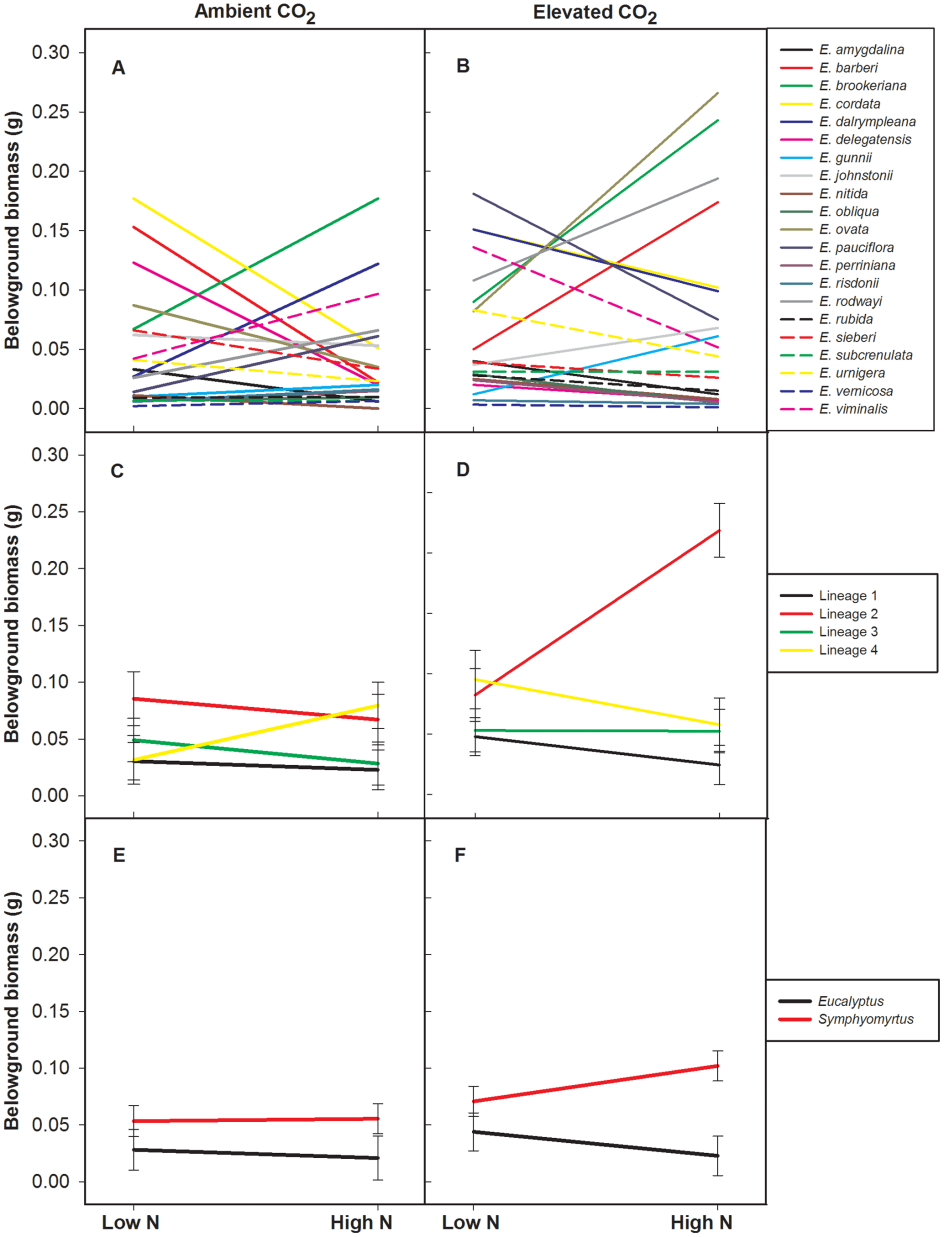
Belowground biomass responses to elevated CO_2_ and N. Interaction plots of least squares means (± standard error for C–F) of the belowground biomass of species (A and B), genetic lineages (C and D) and subgenera (E and F) with control N and 30 kg ha^−1^ of N added (High N) under both ambient (left panels) and elevated CO_2_ (right panels). Standard errors are not presented on the species panels (A–B) due to space constraints. Each colour and line (solid/dashed) combination represents each species, genetic lineage or subgenus.

Similar to aboveground biomass there was also a three-way interaction for belowground biomass. Species belowground biomass displayed a variety of responses to elevated CO_2_ and soil N, whereby species responded differently to soil N availability depending on the concentration of CO_2_ (**Figure 2A, B**). For example, under ambient CO_2_, N addition resulted in a six-fold decrease in the belowground biomass of *E. barberi* whereas under elevated CO_2_, N addition resulted in a 248% increase in belowground biomass. In contrast, N addition under ambient CO_2_ resulted in a 252% increase in the belowground biomass of *E. cordata* but a decrease of 48% under elevated CO_2_. It is clear that individual species responses to the treatment factors were highly variable.

### Phylogenetic Analyses

Similar to the species level analysis, a three-way interaction of genetic lineage, CO_2_ and soil N was identified in both above- and belowground biomass, indicating the importance of evolutionary history in response to these climate change factors. Both the above- and belowground biomass of genetic lineages responded differently to N availability depending on the concentration of CO_2_ (**Table 3** **;** **Figure 1C, D** **;** **Figure 2C, D**). Under ambient CO_2_, above- and belowground responses by genetic lineage did not significantly differ, regardless of N availability. However, under elevated CO_2_, the response of GL2 to N addition significantly differed from other genetic lineages, with large increases in above- and belowground biomass of 213% and 166%, respectively. These results indicate that the response of genetic lineages to CO_2_ and N are highly variable, but they also show that closely related species respond similarly, providing significantly more predictive ability than individual species responses.

**Table 3:** Mixed model results of variation in aboveground biomass (AGB), belowground biomass (BGB), total biomass (TB) and root:shoot (R:S) between the factors genetic lineage, carbon dioxide (CO_2_), nitrogen (N) and all interactions, using the random term species(genetic lineage) to test the genetic lineage effect (random effect not shown).

	*Genetic lineage*		*CO_2_*		*N*		Genetic lineage*CO_2_		Genetic lineage*N		CO_2_*N		Genetic lineage*CO_2_*N	
Variable														
	F	*P*	F	*P*	F	*P*	F	*P*	F	*P*	F	*P*	F	*P*
**AGB**	2.79_(3,21)_	0.066	3.70_(1,186)_	0.056	7.51_(1,186)_	**0.007**	1.19_(3,186)_	0.316	3.68_(3,186)_	**0.013**	0.65_(1,186)_	0.421	6.93_(3,186)_	**<0.001**
**BGB**	3.62_(3,21)_	**0.030**	9.17_(1,183)_	**0.003**	0.22_(1,183)_	0.642	1.12_(3,183)_	0.342	1.49_(3,183)_	0.219	0.14_(1,183)_	0.710	6.50_(3,183)_	**<0.001**
**TB**	2.68_(3,21)_	**0.037**	4.87_(1,180)_	**0.029**	3.17_(1,180)_	0.077	0.95_(3,180)_	0.418	2.10_(3,180)_	0.102	0.57_(1,180)_	0.451	7.10_(3,180)_	**<0.001**
**R:S**	7.28_(3,21)_	**0.002**	12.28_(1,178)_	**0.001**	4.11_(1,178)_	**0.044**	1.18_(3,178)_	0.319	0.51_(3,178)_	0.678	0.44_(1,178)_	0.510	2.34_(3,178)_	0.075

Bold underlined values are significant and degrees of freedom are denoted as subscript of each F value.

Finally, unlike the genetic lineage level comparison, at the broadest phylogenetic level (subgenus), mixed models found no significant interactive effects of subgenus, CO_2_ and N in seedling morphological responses (**Figure 1E, F** **;** **Figure 2E, F**), but there were, however, main effects (**Table 4**). Surprisingly, no significant effects of subgenus, CO_2_ or N were revealed in aboveground biomass. However, there was a significant effect of subgenus on belowground biomass and root:shoot, whereby species in the subgenus *Symphyomyrtus* had a significantly larger belowground biomass (143%) and root:shoot (33%) than species from subgenus *Eucalyptus*. Significant main effects of CO_2_ were also found in belowground biomass and root:shoot indicating that the two subgenera responded to elevated CO_2_ in a similar manner.

**Table 4:** Mixed model results of variation in aboveground biomass (AGB), belowground biomass (BGB), total biomass (TB) and root:shoot (R:S) between the factors subgenus, carbon dioxide (CO_2_), nitrogen (N) and all interactions, using the random term species(subgenus) to test the subgenus effect (random effect not shown).

	*Subgenus*		*CO_2_*		*N*		Subgenus*CO_2_		Subgenus*N		CO_2_*N		Subgenus*CO_2_*N	
Variable														
	F	*P*	F	*P*	F	*P*	F	*P*	F	*P*	F	*P*	F	*P*
**AGB**	3.69_(1,24)_	0.067	1.66_(1,195)_	0.199	2.77_(1,195)_	0.097	0.59_(1,195)_	0.442	2.29_(1,195)_	0.132	0.02_(1,195)_	0.881	2.02_(1,195)_	0.157
**BGB**	5.38_(1,24)_	**0.029**	5.21_(1,192)_	**0.024**	0.02_(1,192)_	0.881	0.32_(1,192)_	0.571	2.02_(1,192)_	0.157	0.03_(1,192)_	0.853	0.83_(1,192)_	0.363
**TB**	4.09_(1,24)_	0.054	3.01_(1,189)_	0.084	0.92_(1,189)_	0.338	0.15_(1,189)_	0.700	2.08_(1,189)_	0.151	0.01_(1,189)_	0.965	0.98_(1,189)_	0.324
**R:S**	7.34_(1,24)_	**0.012**	5.88_(1,187)_	**0.016**	0.84_(1,187)_	0.360	0.02_(1,187)_	0.891	0.37_(1,187)_	0.720	0.720_(1,187)_	0.396	0.65_(1,187)_	0.422

Bold underlined values are significant and degrees of freedom are denoted as subscript of each F value.

## Discussion

### Species-level Response

Independently, elevated CO_2_ or soil N fertilization generally enhances plant growth (e.g. [25], [26], [29], [38], [39]) and two-way interactions between CO_2_ and N availability are also commonly reported in the literature [2], where N availability is expected to constrain the CO_2_-induced stimulation of plant growth [6]. These interactions have been observed across a broad range of tree species from different families and ecological contexts [25], [38], [39], [40], where studies suggest that tree species allocate C to belowground sinks to alleviate N limitation [2]. However in the present study, species displayed considerable variation in root:shoot in response to CO_2_ concentration. Variable responses in biomass allocation to elevated CO_2_ may aid in explaining the variable responses of species to both CO_2_ and N fertilization. Species that display inherently higher proportions of belowground biomass may more effectively exploit soil N and, therefore, respond more strongly to elevated CO_2_ [2]. Evolutionary history and local adaptation are likely to be drivers of these differential responses, indicating the presence of evolutionary trade-offs [9] in the responsiveness of species to either elevated CO_2_ or soil N. A number of species did not appear to respond to elevated CO_2_ and N fertilization whereas, due to variability in traits (e.g. larger root:shoot [41]), others did respond to these factors, either singularly or in combination (interactive effects).

The large variation among species responses is likely to complicate our general understanding of the impacts of global change. For example, Spinnler *et al.* [42] found differential impacts of elevated CO_2_ on tree species within model spruce-beech (*Fagus sylvatica*-*Picea abies*) ecosystems growing on either acidic or calcareous soils with either standard or increased nutrient availability. The biomass of *P. abies* was enhanced by elevated CO_2_ on both soils, whereas *F. sylvatica* only responded to elevated CO_2_ when grown in acidic soil. In this case, the biomass of *F. sylvatica* decreased by 10% with added nutrients and by a further 24% with no added nutrients in response to elevated CO_2_. The results of our study indicate that variable species responses to global change may in fact lead to changes in ecosystem dynamics. The highly variable responses among species within this study suggest that species shifts in eucalypt dominated communities could occur under elevated CO_2_ and changes to soil N due to fertilizer runoff or deposition. These variable species responses also indicate that taking a phylogenetic perspective may provide the generality required to more efficiently predict the growth responses of species to global climate change, rather than assessing the responses of multiple species individually.

### Phylogenetic Similarity in an Ecosystem Response

This study is among the first, to our knowledge, to examine the growth responses of any taxa to global change within a phylogenetic context (but see [11], [13]–[15] for non-growth responses). We found a strong, shared response in plant performance within GL2. Species responses to elevated CO_2_ and soil N were determined by genetic lineage, where phylogenetically shared traits resulted in similar species responses within this group. Similar to the species level analysis, a number of genetic lineages did not appear to respond to elevated CO_2_ and N, whereas due to phylogenetically shared traits (e.g. larger root:shoot), GL2 responded strongly to elevated CO_2_ and soil N fertilization.

Genetic lineage level analyses showed that GL2 responded significantly differently to elevated CO_2_ and N than other genetic lineages; these species demonstrated strong, similar responses to elevated CO_2_ and N. The root:shoot differed significantly among genetic lineages, where GL2 displayed, on average, a 50% greater root:shoot. This shared trait possibly allowed for greater nutrient utilisation, thus stimulating the CO_2_ fertilisation effect [6]. As these species in GL2 generally inhabit nutrient poor areas [32], this trait may have evolved in response to these environmental conditions.

The shared responses of GL2 to elevated CO_2_ and N, suggests that the growth responses of species to the global change factors of elevated CO_2_ and N may be phylogenetically biased, and a result of shared phylogenetic traits among closely related species [13], [14]. Therefore, under a competitive environment containing a number of phylogenetic lineages, there may be ‘winner’ lineages and ‘loser’ lineages. For example, under elevated atmospheric CO_2_ concentrations and high soil N availability, in communities containing species from both GL1 and GL2, species from GL2 may outperform those from GL1. Willis *et al.* [43] applied similar phylogenetic methods to explain the invasiveness of non-native plant groups in Concord, MA (USA), where non-native groups displayed a greater ability to adjust flowering time in response to climate change; flowering time was postulated to be linked to fitness through ecological mismatches such as pollination. The results of our study indicate that a phylogenetic approach may provide a mechanism whereby highly idiosyncratic species-specific responses to elevated CO_2_ and N fertilization may be generalised across closely related species, perhaps providing a better understanding of the effects of global change on ecosystem dynamics.

When species responses were analysed for the effects of subgenus, the subgenera differed in belowground biomass and root:shoot but did not significantly interact with CO_2_ or N. These results indicate that there is a phylogenetic effect and the two subgenera respond to CO_2_ and N fertilization in the same way; thus this level of phylogeny may be too broad to differentiate effects of CO_2_ and N on morphological traits. Overall, these results suggest that more recently evolved traits at the lower phylogenetic grouping (genetic lineage) level are likely to be responsible for the large proportion of variation among species in responses to elevated CO_2_ and N fertilization.

### Conclusions and Implications

Predictions of future plant distributions and the sustainability of the services those ecosystems provide are often made using niche-based models that implement correlative methods that relate the presence or absence of species across environmental gradients [44], [45]. These models may be species-specific or generalised over species with similar fundamental niches. Using species-specific responses to both elevated CO_2_ and N may not be the ideal tool to use for predicting the responses of ecosystems to global environmental change [45], for conservation or climate change mitigation. The results of this study suggest that the responses of forest trees to elevated CO_2_ and N may not be random, but phylogenetically biased. A phylogenetic approach may provide a possible alternative to species-specific studies since it could be applied across many plant groups and ecological contexts. To consolidate our findings, further studies are warranted. For example, field studies would determine if phylogenetic patterns in species responses persist while subject to natural conditions. Understanding the evolutionary relationships of species may help us better understand and predict the future ecology of forest communities and as phylogenetic information continues to become exponentially more available, its utilization could complement the plant functional group approaches that are currently represented in many dynamic global vegetation models [46], [47]. However, this raises the question of which phylogenetic level should be chosen to best predict species responses? The utilisation of the phylogenetic level which most adequately represents the distribution of the climatically relevant traits would be most pertinent.
